# Non-Invasive Methods for Predicting the Quality of Processed Horticultural Food Products, with Emphasis on Dried Powders, Juices and Oils: A Review

**DOI:** 10.3390/foods10123061

**Published:** 2021-12-09

**Authors:** Emmanuel Ekene Okere, Ebrahiema Arendse, Helene Nieuwoudt, Olaniyi Amos Fawole, Willem Jacobus Perold, Umezuruike Linus Opara

**Affiliations:** 1 SARChI Postharvest Technology Research Laboratory, Africa Institute for Postharvest Technology, Faculty of AgriSciences, Stellenbosch University, Stellenbosch 7600, South Africa; 22093427@sun.ac.za (E.E.O.); arendse@sun.ac.za (E.A.); 2 Department of Electrical and Electronic Engineering, Stellenbosch University, Private Bag X1, Matieland 7602, South Africa; wjperold@sun.ac.za; 3 Department Viticulture and Oenology, Institute for Wine Biotechnology, Stellenbosch University, Private Bag X1, Matieland 7602, South Africa; hhn@sun.ac.za; 4 Postharvest Research Laboratory, Department of Botany and Plant Biotechnology, University of Johannesburg, P.O. Box 524, Auckland Park, Johannesburg 2006, South Africa; olaniyif@uj.ac.za; 5 UNESCO International Centre for Biotechnology, Nsukka 410001, Nigeria

**Keywords:** infrared spectroscopy, hyperspectral imaging, X-ray micro-computed tomography, nuclear magnetic resonance, Raman spectroscopy, juice quality, oil quality, powder quality

## Abstract

This review covers recent developments in the field of non-invasive techniques for the quality assessment of processed horticultural products over the past decade. The concept of quality and various quality characteristics related to evaluating processed horticultural products are detailed. A brief overview of non-invasive methods, including spectroscopic techniques, nuclear magnetic resonance, and hyperspectral imaging techniques, is presented. This review highlights their application to predict quality attributes of different processed horticultural products (e.g., powders, juices, and oils). A concise summary of their potential commercial application for quality assessment, control, and monitoring of processed agricultural products is provided. Finally, we discuss their limitations and highlight other emerging non-invasive techniques applicable for monitoring and evaluating the quality attributes of processed horticultural products. Our findings suggest that infrared spectroscopy (both near and mid) has been the preferred choice for the non-invasive assessment of processed horticultural products, such as juices, oils, and powders, and can be adapted for on-line quality control. Raman spectroscopy has shown potential in the analysis of powdered products. However, imaging techniques, such as hyperspectral imaging and X-ray computed tomography, require improvement on data acquisition, processing times, and reduction in the cost and size of the devices so that they can be adopted for on-line measurements at processing facilities. Overall, this review suggests that non-invasive techniques have the potential for industrial application and can be used for quality assessment.

## 1. Introduction

The consumption of fresh horticultural produce plays an important role in human nutrition and health, as it is known to be a major source of major and minor macro-nutrients, antioxidants, and dietary fibre [1]. Most fruits and vegetables are highly perishable due to their high water content, resulting in post-harvest losses throughout the value chain. Therefore, fresh fruits and vegetables are processed into value-added products to extend the shelf-life, minimize food waste, and preserve the nutritional quality of horticultural produce. The main objective of processing is to extend the shelf-life of perishable products and preserve their nutritional, textural, and sensory properties [2]. Consequently, during post-harvest processing, storage, and transportation, the physiological quality of processed products continues to change. As a result, it is important to monitor the quality and food safety of processed products throughout the value chain [3]. Food-processing chains undergo several steps which pre-expose them to pathogenic infestation, adulteration, and contamination with unwanted chemical compounds, whether deliberately or accidentally acquired. During processing and throughout the value chain, products, such as juices, oils, and powders, are often at high risk of adulteration with cheaper substitutes. This safety concern with pre-exposure of the processing value chain poses a serious health risk to humans and consumers and affects the sensory perception of products, reducing the nutritional value and functional properties of the processed commodity. Accordingly, it is highly important and necessary that processed horticultural products, such as oils, powders, and juices, are subjected to stringent inspection for quality and authenticity.

The global food processing industry is frequently confronted by new technological challenges to meet the increasing demand for quality-assured processed products. Consequently, over the last decade, the food processing industry has shifted the way it performs quality measurement from subjective assessment to the widespread adoption of objective non-invasive technologies that provide faster and more reliable results. These non-invasive technologies are now commercially available for quality control as either small desktop or online grading units. Several studies have highlighted the potential of different non-invasive approaches and methods applied to processed horticultural products and have promoted its use as a rapid and non-invasive analytical method for quantitative or qualitative analysis [4,5,6,7]. These include infrared spectroscopy [8], hyperspectral and multispectral imaging [9,10], Raman spectroscopy [11], nuclear magnetic resonance (NMR) [12], and X-ray computed tomography [13]. Several of these techniques have been successfully employed in classification, authenticity, and quantification in commercial juices, oils, powders, and dried products [14].

Currently, scientific literature and reviews on various non-destructive technologies for the quality measurement of fresh fruit and vegetables are abundant. However, there is a dearth of literature reviews on the non-invasive measurement of processed horticultural products. A refined Scopus search over the last decade revealed that there are currently 59 reviews for non-destructive assessment of fresh fruit and vegetable quality; only four reviews included information on aspects of some processed horticultural products [1,15,16]. Additionally, the reviews with information on some processed horticultural products mainly focus on assessing either a specific product or non-destructive technique and do not integrate assessment of different products or review other possible non-destructive techniques. Due to the shortage of scientific information on non-invasive quality assessment of different processed horticultural products, this review can provide readers with insight into the current usage of non-invasive methods, highlighting a potential research scientific gap. This review aims to provide a summary of the recent developments in non-invasive technologies for the quality assessment of different processed horticultural products with an emphasis on juices, oils, and powdered products.

### 1.1. The Concept of Quality

The word “quality” is derived from the Latin “qualitas”, which means attribute, property, or basic nature of an object [17]. Several experts within the field have proposed brief definitions of quality, e.g., “fitness for use” [18], “conformance to requirements” [19], “loss avoidance” (Taguchi, cited in [20]), “degree of excellence or superiority” [21], with a view to understanding and optimizing the whole system of value exchange [22,23]. The various contexts of use have generated multiple definitions depending on the field of study and the term has been used to describe various phenomena. For the agricultural industry, the quality of fresh and processed horticultural produce is assessed based on a combination of values for several attributes or characteristics specific to the commodity. The quality attributes of fresh and processed horticultural crops comprise a combination of sensory characteristics (e.g., appearance, texture, flavour, and aroma), nutritional values, bio-chemical constituents, textural properties, and the presence or absence of defects [24]. Therefore, it is difficult to decide on a single universal definition of quality regarding horticultural products considering the different views on quality held by major stakeholders in the field of horticulture [25,26].

### 1.2. Quality Measurement and Evaluation

**Figure 1:**
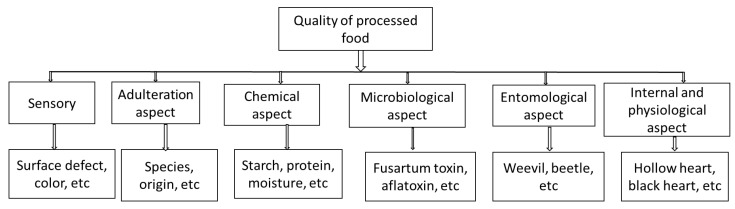
A block diagram of different food quality aspects and related parameters [24,25,26,29].

The measurement of the quality attributes of horticultural produce plays an important role in quality management during post-harvest handling. These measurements allow for comparison against industrial standards by ensuring that the product meets the limits of acceptability by the consumer [27]. Product quality attributes may be evaluated using a sensory panel or instrumental analysis. The quality attributes of various agricultural products are often defined by a combination of properties, such as physical, chemical, and microbial characteristics [16]. These quality attributes include appearance (e.g., size, shape, gloss and colour, freedom of defect and decay), texture (e.g., firmness, crispness, juiciness, and mealiness), flavour (e.g., sweetness, acidity, astringency, aroma, and off-flavours), and nutritive value (e.g., dietary fibre, vitamins, minerals, and phytonutrients) [6,28,29]. Other measurable quality attributes such as fat content, moisture, and protein content, are also analysed [30]. Figure 1 shows a block diagram of different food quality aspects and related parameters. The upper line indicates the different food quality aspects, while the bottom line indicates the different attributes of the different aspects of quality.

### 1.3. Parameters Used for Evaluating Models’ Performance

For non-invasive quality analysis, the performance criteria for developing multivariate models can be assessed based on different factors of merit. One of the most used criteria for the assessment of a model’s performance includes the coefficient of determination (*R*^2^), for prediction (*R*^2^p), calibration (*R*^2^c), and cross-validation (*R*^2^cv). Other statistical parameters that can be used to assess a model’s performance include the root mean square error (RMSE) for calibration (RMSEC), prediction (RMSEP), and cross-validation (RMSECV), residual predictive deviation (RPD), and bias [1,31,32].

## 2. Infrared Spectroscopy

### 2.1. Overview of Infrared Spectroscopy

**Figure 2:**
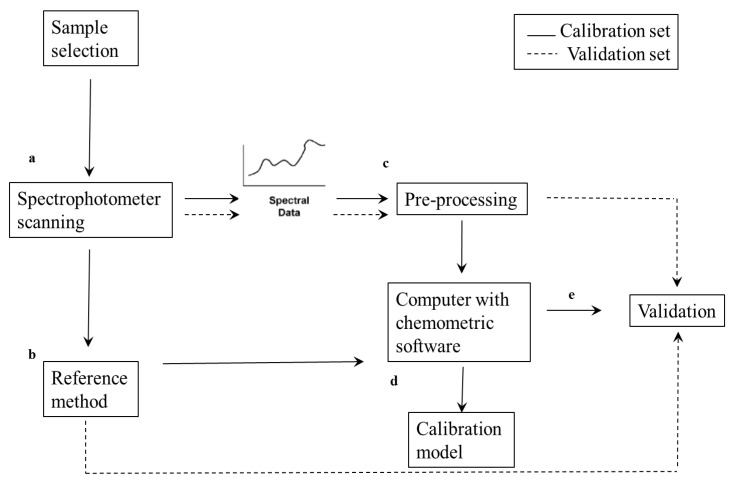
Block diagram with basic steps for developing NIR calibration model. (**a**) the sample is irradiated with NIR radiation, (**b**) a fundamental analytical method also known as reference method is used to obtain the dependant variable to be calibrated, (**c**) the acquired spectral data is subjected to pre-processing methods using chemometrics, (**d**) the combination of reference and spectral data are used to develop calibration model, (**e**) calibration models are validated to test the model performance.

Infrared (IR) spectroscopy is the most commonly used non-invasive technique and utilizes the interaction of molecular bonds and electromagnetic radiation within the near-infrared (NIR, 12,500–4000 cm^−1^ or 800–2500 nm) and mid-infrared (MIR, 4000–400 cm^−1^ or 2500–25,000 nm) ranges, respectively. The NIR spectrum of a biological sample consists of broad overlapping bands that correlate to the vibration of chemical bonds such as C–H, O–H, and N–H within a molecule. This results in the development of broadband overtones. Compared to other non-invasive techniques, NIR spectroscopy (NIRs) has several advantages. For instance, NIRs can penetrate food samples at a finite distance and provide spectral information on the physical and chemical characteristics of a biological sample [8]. MIR spectroscopy (MIRs) provides more spectral data and has a higher spectral resolution compared to NIRs; this is because the MIR spectrum contains wavelength ranges for molecular vibration that are highly sensitive to various chemical compositions within a biological sample [33,34]. A block diagram illustrating the basic steps of NIR spectral acquisition and model development is presented in Figure 2.

### 2.2. Application of Infrared Spectroscopy for Assessment of Processed Horticultural Products

#### 2.2.1. Dried Horticultural Products

The quality standards of dried horticultural products should have specific standards before they are considered safe for consumption. These include microbial load, moisture content, particle size, and particle morphology. Attention needs to be given to the quality evaluation of powdery food particles, with emphasis on parameters such as chemical composition (e.g., regarding starch, protein), adulteration, and mycotoxin content. Recent progress and applications of IR spectroscopic technologies for the non-invasive quality determination of powdery horticultural products are reviewed (Table 1). For the evaluation of tea powder, Fourier-transform near infrared (FT-NIR) spectroscopy provided a satisfactory performance for the prediction of catechin, with prediction statistics (*R*^2^ = 0.921–0.971, RMSEP = 0.017–0.384%) [35]. MIR spectroscopy was applied by Li et al. [36] to detect polyphenols in tea powder. The authors combined the wavelength selection algorithms of backward interval partial least squares (biPLSR) in this study. Comparable results were obtain using partial least square regression (PLSR) (*R*^2^ = 0.708) and biPLSR (*R*^2^ = 0.713). In a different study, Li et al. [37] investigated FT-MIR spectroscopy to quantify talcum powder contamination in tea powder, based on a hybrid of biPLSR, competitive adaptive reweighted sampling (CARS), and selection of 18 characteristic variables. The authors obtained better detection results of talcum concentration (*R*^2^ = 0.927, RMSEP = 0.137). Other physicochemical properties that were evaluated using IR spectroscopy have included starch [38], protein [39], metanil yellow [40,41], and total phenolic content [42]. Coffee is another dried horticultural product that is globally consumed. Recent studies have shown that FT-NIR spectroscopy can assess the quality characteristics of ground coffee. For instance, Tugnolo et al. [43] evaluated an in-line FT-NIR system to help characterize ground coffee. The authors reported that FT-NIR spectroscopy can predict moisture content (%) (*R*^2^ = 0.980, RPD = 8.0), tap density (g/L) (*R*^2^ = 0.700, RPD = 1.8), and powder granulometry (%) (*R*^2^ = 0.920, RPD = 3.5). Adnan et al. [44] used FT-NIR spectroscopy and applied linear discriminant analysis (LDA) to distinguish between two varieties of ground coffee (Arabica and Robusta). The discrimination of the ground coffee was based on caffeine and chlorogenic acid contents. The authors reported a classification accuracy of 95.5%. In a more recent study, Tugnolo et al. [45] applied partial least squares regression (PLSR) models using FT-NIR spectroscopy and observed high predictive performances (*R*^2^ = 0.970 and RMSEC = 0.13%) for Arabica and Robusta ground coffee varieties.

IR spectroscopy has been successfully used in the detection and classification of mycotoxins and the adulteration of powdered foods. For instance, Tripathi and Mishra [4] used FT-NIR spectroscopy to detect Aflatoxin B_1_ in chilli powder. The authors reported that model development using PLSR provided satisfactory results with *R*^2^ of 0.967 and RMSECV of 0.654%. For adulteration, Haughey et al. [46] used NIR spectroscopy to detect fraudulent adulteration of chilli powder using Sudan dye (0.1–5%). The developed quantitative models determined that the limit of detection was 0.25%, and coefficients of determination (*R*^2^) were found to be 0.991–0.994. In a similar study, Hu et al. [47] identified the adulteration of Sichuan pepper using Fourier transform mid-infrared spectroscopy and tested the authenticity of black pepper using several algorithms. They observed that when using both the genetic algorithm and support vector machine (GA-SVM) and partial least squares-discriminant analysis (PLS-DA), the developed models attained a 100% classification rate. These studies have reported successful applications of both FT-NIRs and FT-MIRs in different horticultural dried or powdered products, suggesting that the techniques can be further explored for the quality assessment of other dried fruit samples, such as dried pomegranate arils, banana slices, and others.

**Table 1:** Summary of infrared spectroscopy applied for quality evaluation of different horticultural dried products.

Products	Non-Invasive Method	Parameters	Wavelength Range	Predictors Accuracy	References
Black tea	NIRs	Caffeine Free amino acid Total phenolics Water extract	800–2500 nm	*R*^2^ = 0.955, RMSEP = 0.16% *R*^2^ = 0.927, RMSEP = 0.273% *R*^2^ = 0.954, RMSEP = 0.594% *R*^2^ = 0.962, RMSEP = 0.685%	[42]
Chilli powder	NIRs	Aflatoxin B_1_	12,000–4000 cm^−1^	*R*^2^ = 0.967, RMSECV = 0.654%	[4]
Chilli powder	NIRs	Sudan I dye	9000–4000 cm^−1^	*R*^2^ = 0.991, RMSEP = 0.141%	[46]
Corn flour	NIRs	Protein	10,000–4000 cm^−1^	*R*^2^ = 0.882, RMSEP = 0.413%	[39]
Garlic powder	MIRs	Starch	4000–650 cm^−1^	*R*^2^ = 0.950 for VIP, *R*^2^ = 0.890 for SR	[38]
Coffee powder	NIRs	Moisture content	960–1650 nm	*R*^2^ = 0.980, RMSECV = 0.02%, RPD = 8.0	[43]
		Tap density	960–1650 nm	*R*^2^ = 0.700, RMSECV = 13.70 g/L, RPD = 1.8	
		Powder granulometry	960–1650 nm	*R*^2^ = 0.920, RMSECV = 1.23%, RPD = 3.5	
Coffee powder	NIRs	Moisture content	960–1650 nm	*R*^2^ = 0.970, RMSEP = 0.13%	[45]
Lotus root flour	MIRs	Starch	4000–500 cm^−1^	*R*^2^ = 0.981, SDR = 5.47%	[40]
Tea powder	MIRs	Catechin	4000–1000 cm^−1^	*R*^2^ = 0.921–0.971, RMSEP = 0.017–0.384%	[35]
Tea powder	MIRs	Polyphenol	4000–400 cm^−1^	*R*^2^ = 0.708–0.713	[48]
Tea powder	MIRs	Talcum concentration	4000–400 cm^−1^	*R*^2^ = 0.927, RMSEP = 0.137%	[37]
Turmeric powder	MIRs	Metanil yellow	4000–650 cm^−1^ 3700–100 cm^−1^	Detection of 5% (*w*/*w*) 1% (*w*/*w*)	[41]

RMSEP, root mean square error of prediction; NIRs, near-infrared spectroscopy; MIRs, mid-infrared spectroscopy; RMSECV, root mean square error of cross-validation; *R*^2^, coefficient of determination; VIP; variable importance in projection; SR; selectivity ratios.

#### 2.2.2. Juice Products

Over the last decade, several research publications on the usage of IR spectroscopy for quality control, and authenticity of commercial juices, were published (Table 2). Xie et al. [49] applied NIRs to determine individual sugars (e.g., glucose, fructose, sucrose) in bayberry juice in the NIRs region of 800–2400 nm. According to Włodarska et al. [50], the NIRs region of 6896, 5587, and 4413 cm^−1^ was optimal for assessing sucrose, fructose, and glucose in apple juice. For Satsuma mandarin juice, Masithoh et al. [51] investigated the usage of visible to near-infrared spectroscopy (Vis/NIRs) (600–1100 nm) to measure the soluble solid content (SSC) and titratable acidity (TA). The authors reported calibration models predicting SSC with *R*^2^ of 0.92 °Brix, standard error of prediction (SEP) = 0.42, and acidity with *R*^2^ = 0.56, SEP = 0.14%.

In a recent study, Cassani et al. [52] investigated the usage of FT-MIR spectroscopy to simultaneously quantify simple sugars and exogenously added fructo-oligosaccharides (FOS) in 4 types of strawberry juices during storage. The authors performed principal component analysis (PCA), which explained (76–97%) of the variation and observed an *R*^2^ of 0.97 for the developed PLS models. Ayvaz et al. [53] applied FT-MIR spectroscopy in the spectral region of 1460–950 cm^−1^ for predicting glucose, fructose, total reducing sugars, soluble solids (°Brix), and serum viscosity in tomato juice. Using PLS regression analysis the authors predicted glucose (R_pred_ = 0.95, SEP = 1.4 g/L), fructose (R_pred_ = 0.95, SEP = 1.46 g/L), total reducing sugars (R_pred_ = 0.97, SEP = 2.06 g/L), soluble solids (°Brix) (R_pred_ = 0.99, SEP = 0.12 g/L) and serum viscosity (R_pred_ = 0.85, SEP = 1.32) with good accuracy. MIRs have successfully been applied to evaluate the quality of several juices, including grape [54], tomato [55], blackcurrant [56], sugar beet juice [57], mango [58], and pomegranates [59].

From the published literature, MIR spectroscopy seems more suitable for the evaluation of juice quality. This is because the MIR spectrum consists of wavelength ranges that are highly sensitive to various chemical constituents compared to the broadband overtones in the NIR region. For comparison between NIR and MIR spectroscopy, Caramês et al. [60] conducted a study to develop calibration models to predict total phenolic content (TPC) and total anthocyanin content (TAC) in grapefruit. In their study, PLSR algorithms were applied to develop prediction models, and the results showed that MIRs (RMSEP = 4.44 mg/100 mL) and NIRs (RMSEP = 4.22 mg/100 mL) both predicted the TAC with similar accuracy. However, in the prediction of TPC, MIRs provided a lower RMSEP (0.21 mg GAE/mL) value which provided slightly better results compared to that of the NIRs (0.37 mg GAE/mL). This indicates that it is easier to derive information from the MIR spectral region than the NIR spectral region. In another study, Arendse et al. [59] statistically compared the usage of several NIR and MIR instruments for the quality evaluation of pomegranate juices. The authors observed that MIRs performed better in the prediction of quality parameters such as TSS, TA. However, their statistical approach based on Bland and Altman, and Passing-Bablok suggested that both NIRs and MIRs showed no significant differences between the two instruments.

For classification and authentication of adulterated juices, Xie et al. [61] reported that NIRs can classify and discriminate bayberry juice adulterated with water. By using radial basis function neural network classifiers, the authors acquired a classification accuracy of 97.62%. Šnurkovič [62] demonstrated discrimination between a mixture of various juices that were adulterated with water, sugar, and fruit juices which was based on NIR spectral data. Several other investigators reported that infrared spectroscopy, specifically the MIR spectral region, has successfully been applied for authentication and adulteration testing [14,58,63]. Table 2 gives a summary of the application of IR spectroscopy for juice samples of horticultural fruit. 

**Table 2:** Summary of infrared spectroscopy applied for quality evaluation of different horticultural juice products.

Product	Non-Invasive Method	Parameters	Wavelength Range	Predictors Accuracy	References
Apple juice	NIRs	SSC TA SSC/TA	12,500–4000 cm^−1^	*R*^2^ = 0.881, RMSECV = 0.277% *R*^2^ = 0.761, RMSECV = 0.239% *R*^2^ = 0.843, RMSECV = 0.113%	[50]
Bayberry juice	NIRs	Glucose Fructose Sucrose	800–2400 nm	*R*^2^ = 0.746–0.854 *R*^2^ = 0.698–0.963 *R*^2^ = 0.890–0.993	[49]
Black currant juice	MIRs	SSC TA	7000–600 cm^−1^	*R*^2^ = 0.97, RMSECV = 1.14% *R*^2^ = 0.96, RMSECV = 2.61%	[56]
Grape juice	Vis/NIRs	SSC pH	325–1075 nm	*R*^2^ = 0.979, RPD = 6.971 *R*^2^ = 0.951, RPD = 5.432	[54]
Grape juice	MIR/NIR	TAC TPC	10,000–829.11 cm^−1^ 10,000–823.52 cm^−1^	*R*^2^ = 0.81, RMSEP = 4.22–4.44 mg/100 mL *R*^2^ = 0.90, RMSEP = 0.21–0.37 GAE mg/100 mL	[60]
Mango juice	MIRs	ASC TSS RJC	4000–650 cm^−1^	*R*^2^ = 0.996 *R*^2^ = 0.997 *R*^2^ = 0.986	[58]
Pomegranate juice	NIRs/MIRs	TSS TA TSS/TA	12,500–4000 cm^−1^	*R*^2^ = 0.923, RMSEP = 0.31%, RPD = 3.63 *R*^2^ = 0.862, RMSEP = 0.11%, RPD = 2.7 *R*^2^ = 0.817, RMSEP = 1.04%, RPD = 2.35	[59]
Strawberry juice	MIRs	Glucose, sucrose, fructose	1200–900 cm^−1^	*R*^2^ ≥ 0.97	[52]
Satsuma mandarin	Vis/NIRs	SSC TA	600–1100 nm	*R*^2^ = 0.92, SEP = 0.42 °Brix *R*^2^ = 0.56, SEP = 0.14%	[51]
Tomato juice	NIRs	SSC pH	800–2400 nm	100% accuracy	[52]
Tomato juice	MIRs	Glucose, fructose, TSS, viscosity	1460–950 cm^−1^	R_pred_ ≥ 0.82	[53]

*R*^2^, coefficient of regression; RMSECV, root mean square error of cross-validation; RMSEP, root mean square error of prediction; RPD, residual predictive deviation; R_pred_, correlation coefficient for prediction; NIRs, near-infrared spectroscopy; Vis/NIRs, visible to near-infrared spectroscopy; MIRs, mid-infrared spectroscopy; TSS, total soluble solids; SSC, soluble solid content; TA, titratable acidity; TPC, total phenolic content; TAC, total anthocyanin content; ASC, added sugar content; RJC, real juice content.

#### 2.2.3. Oil Products

For quality control in edible fats and oils, oxidation is one of the main parameters used for oil products. Oxidation of fats and oils produces either primary (peroxides) or secondary oxidation products. Recently, FT-IR spectroscopy, in combination with chemometrics, has been used to predict the occurrence of oxidation of a variety of oil products (Table 3). Marina et al. [64] applied 30 samples of virgin coconut oil for the quantitative analysis of peroxide value using Fourier transform infrared (FT-IR) spectroscopy. In their study, calibration models were obtained that yielded satisfactory results with an RMSEP value of 0.497 and an *R*^2^ of 0.982. In a similar study, Marina et al. [65] investigated the free fatty acid profile of virgin coconut oil using FT-MIR spectroscopy in the spectral region of 1730–1690 cm^−1^. The authors obtained satisfactory results with *R*^2^ = 0.928 and RMSEP = 0.126. The results illustrated the great potential of FTIR application in the rapid, accurate quantification of virgin coconut oil. In another study, Mba et al. [66] applied MIRs to assess the quality of palm and canola oil. The authors achieved satisfactory results for iodine value (IV), free fatty acid (FFA), and peroxide value with *R*^2^ values ≥ 0.98 and RPD value ranging from 6.11–11.60. Other quality parameters of different edible oils have been evaluated using IR spectroscopy. These include acid value [67], peroxide value [5,68], total phenolic content [67], squalene [69], and total sterol content [70].

Researchers have also compared the performance of NIR and MIR in classification, authentication, and adulteration detection for different horticultural oil products [71,72,73,74]. Their findings demonstrated the ability of this technique to successfully replace the existing traditional wet chemistry analytical methods of quality analysis.

## 3. Hyperspectral Imaging (HSI) and Multispectral Imaging (MSI)

### 3.1. Overview of Hyperspectral Imaging (HSI) and Multispectral Imaging (MSI)

**Figure 3:**
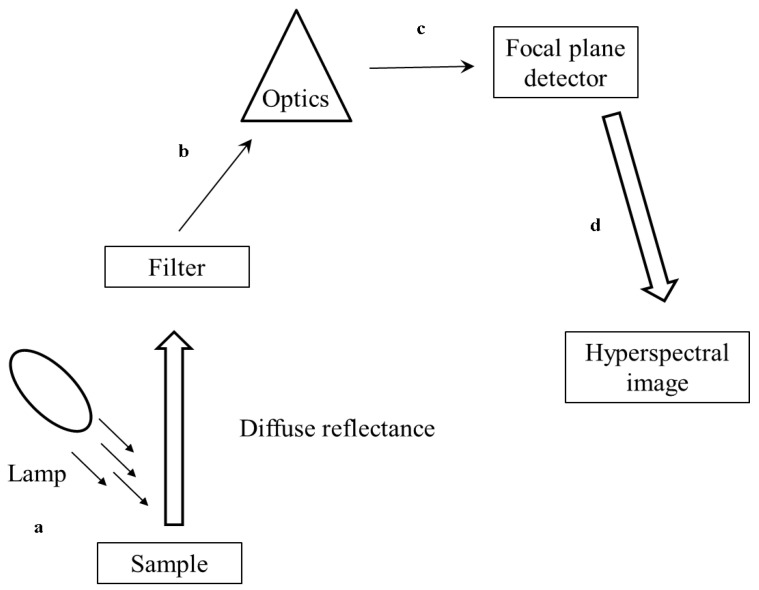
Block diagram of basic steps for hyperspectral imaging. (**a**) The sample is radiated with NIR radiation, (**b**) the reflected radiation is captured by a filter and optics which is responsible for wavelength selection, separation, and measurement, (**c**) the spectrum of each pixel is captured and is recorded by a detector, (**d**) the image of the sample at each wavelength is recorded resulting in image slices.

Hyperspectral imaging (HSI) is a non-invasive technique that integrates spectroscopic and imaging into one system [75]. It is often used to gather images with spatial and spectral data and has been widely used for the studied food technique. HSI simultaneously acquires large amounts of monochromatic images with wavebands, and a full spectrum is extracted for each pixel in an image [1]. The data obtained from HSI systems are 3-dimensional (3D) structures that consist of two spatial and one spectral dimension [1]. The process usually requires a significant amount of time for image acquisition under laboratory conditions and relatively complicated procedures for offline image analysis. On the other hand, multispectral imaging (MSI) is considered a reformation of hyperspectral imaging [75]. It involves creating images using more than one spectral component of the electromagnetic wavelength from the same region of an object and at the same scale [31,76]. MSI aims to acquire spatial and spectral information useful for real-time applications (e.g., in packing houses and food processing plants). MSI accomplishes this by capturing two or more waveband monochromatic images with a few (generally less than 10) discrete wavebands in the spectrum [77]. This process involves fast image acquisition and simple algorithms for image processing and decision making. HSI and MSI make use of one of three methods to generate 3D hyperspectral cubes [hypercubes (x, y, k)]; these include point (whiskbroom) scanning, line (pushbroom) scanning, and area scanning (tunable filter or staredown). A block diagram illustrating the basic steps of HSI and MSI spectral acquisition and model development is presented in Figure 3.

MSI for real-time application is not feasible as the point-scan method may not be suitable for fast image acquisition due to scanning along two spatial dimensions as it makes it time-consuming. Moreover, the previously mentioned methods (i.e., line-scan and area-scan) can be adjusted to meet the requirements for rapid image acquisition. Hyperspectral imaging, when compared to traditional analytical methods, is highly sensitive to minor constituents, but has a poor limit of detection [78]. Further information on the principles of these technologies can be found in a review by Qin et al. [79].

### 3.2. Application of Hyperspectral Imaging (HSI) and Multispectral (MSI) for Assessment of Processed Horticultural Products

#### 3.2.1. Dried Horticultural Products

For dried horticultural products, such as seeds, Wang et al. [80] applied the PLS-DA to distinguish between different varieties of tomato seeds. The authors applied the method on a spectral range of 375–970 nm and reported a classification accuracy of above 82%. In another study, Shrestha et al. [81] also discriminated between tomato seeds using PLS-DA and spectral range. The authors reported a classification accuracy ranging from 94% to 100%. Other applications of HSI have been reported on spinach seed [82] and watermelon seed [83]. For roasted coffee, Caporaso et al. [84] applied HSI to predict its aroma profile using PLS regression over a spectral range of 1000–2500 nm. The authors obtained a total of 21 volatile compounds and successfully predicted key aroma compounds that provided prediction statistics of *R*^2^ ranging from 0.21 to 0.71 and RPD values ranging from 0.84 to 1.87. These compounds included aldehydes and pyrazines. Forchetti & Poppi [85] applied HSI and multivariate curve resolution modelling to detect and quantify adulterants in ground coffee, which was mixed with coffee husks, roasted, and powdered corn kernels. The authors reported that the developed methodology was suitable for quantifying adulterants with mixtures ranging from 1 to 40% (*w*/*w*), resulting in less than 4% prediction error. For the evaluation of powdery foods, research and application of HSI have mainly been applied to the evaluation of agronomy powders, such as soybean flour, wheat flour, and oat flour [86]. The ranges usually considered were: 400–1000, 700–1000, 960–1750, and 1100–2400 nm [1]. These powders have been successfully analysed for colour classification, authenticity, contamination, and mycotoxins.

#### 3.2.2. Juice Products

From the literature search, it can be observed that limited information is available on the application of HSI and MSI to the quality evaluation of horticultural processed juices. The reason for this may be that HSI is a visual technique, and most juices have a homogeneous matrix, therefore, it would be understandable that preference is often given to infrared spectroscopic techniques. By using both spectral and spatial data in one system, HSI can analyse and visualise different food matrices and therefore provide richer information than infrared spectroscopy.

#### 3.2.3. Oil Products

The HSI has also successfully been applied for the classification, authenticity detection, and quality evaluation of edible oils. In a study on near-infrared hyperspectral imaging, Guo et al. [87] used HSI to classify edible oil and waste vegetable oil based on their spectral characteristics within the 350–2500 nm spectral region. The authors successfully classified 22 corresponding types based on clustering their hyperspectral digital numbers by applying the unweighted distance method and interior square sum distance. To evaluate chemical indexes in virgin olive oil, Martinez Gila et al. [88] applied two-component algorithms, namely PLS and genetic algorithm (GA-PLS). The authors reported comparable results for the two algorithms for acidity, peroxide value, and humidity content, with *R*^2^ of 0.95, 0.98, and 0.91, respectively, for PLS, and *R*^2^ of 0.93, 0.92, and 0.92 for GA-PLS. These results suggest that HSI can quickly and simultaneously predict various oil quality parameters. A summary of the different applications of HSI and MSI for quality evaluation of processed horticultural products is provided in Table 4.

## 4. X-ray Micro-Computed Tomography

### 4.1. Overview of X-ray Micro-Computed Tomography

**Figure 4:**
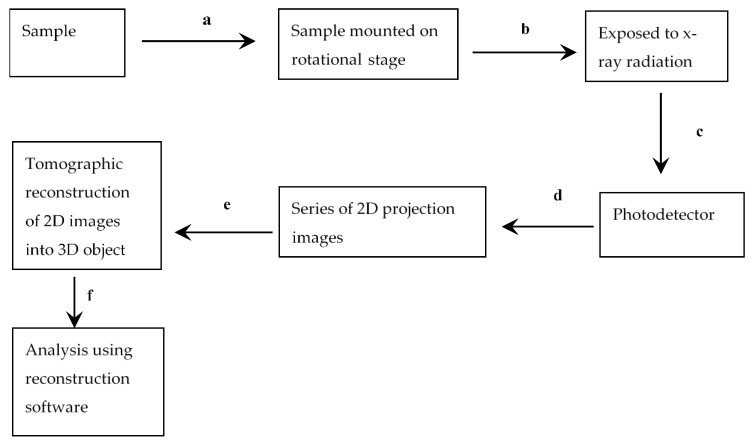
Block diagram of X-ray-CT acquisition and reconstruction process. (**a**) selected sample mounted on a rotational stage, (**b**) a collimated X-ray radiation is focused on sample (**c**) the remainder radiation is captured by a multi-channel detector, (**d**) which transmits a response signal to a computer and produces a series of 2D projection images, (**e**) 2D images are then reconstructed into a 3D object, (**f**) the 3D object is analysed using reconstruction software.

Microfocus X-ray computed tomography (µCT) is a visualization technique that reconstructs and renders three-dimensional images which are used for characterization and defect detection [91,92]. X-ray µCT employs the principle of attenuation of X-rays and measures the variation in the density of the sample [93]. X-ray computed tomography is based on an X-ray radiograph. An X-ray beam is focused on the sample, and the transmitted radiation is recorded by a multi-channel detector. The transmission of the radiation depends on the mass density and mass absorption coefficient of the scanned material. A series of 2-dimensional (2D) images are captured while rotating the sample between 0° and 180° using a filtered back-projection algorithm. The volume of the object can be reconstructed into a 3-dimensional (3D) image which is superimposed on information (projection) of the stack of 2D images [91]. A block diagram illustrating the basic steps of X-ray µCT and image development is presented in Figure 4.

X-ray µCT allows for the visualization, characterization, and analysis of physical and physiological structures of biological materials with a resolution as low as a few micrometres. Considering that fruits and vegetables have a high moisture content, water dominates X-ray absorption and affects the density of a material and, therefore, structures and defects can be visualized [91]. X-ray µCT is one of the most powerful non-invasive techniques for evaluating internal and external characteristics, including the detection of defects in agricultural products [94]. Further information on the principles of these technologies has been explicated and extensively reviewed by Kotwaliwale et al. [92].

### 4.2. Application of X-ray Computed Tomography for Assessment of Processed Horticultural Products

The application of X-rays in the commercial assessment of agricultural commodities is still in the early stages of development. However, studies have shown that X-ray µCT has considerable advantages in defect detection and characterization which complement current inspection techniques [95]. Research studies regarding X-ray µCT have mainly focused on the non-invasive characterization of food microstructure for fresh horticultural fruits (Table 5). However, limited information has been published on horticultural products processed into powders (dried) and seed oil. The application of X-ray is an effective technique for the estimation and characterization of internal structures. For instance, Arendse et al. [96], estimated the juice content of pomegranate fruit cv. “Wonderful” grown in South Africa. The authors predicted the juice volume (142.7 ± 16.4 mL), constituting 89.8% of the total aril volume (162.5 ± 16.2 mL). In a similar study on the same fruit, Arendse et al. [13] successfully quantified various fruit parts using X-ray µCT, namely, fruit juice content, aril volume and number, and kernels. Magwaza and Opara [97] estimated the volume of minimally processed pomegranate arils. The limited knowledge of X-ray µCT applied to dried fruit samples and seed oil provides novel research opportunities to evaluate the quality of these processed horticultural products.

A review of scientific literature shows comprehensive application of X-ray µCT for the non-invasive defect detection and characterization of internal and external structures of biological materials [98,99,100]; however, commercial applications for real-time or on-line detection have been limited. Some of the drawbacks of X-ray µCT systems are that they are expensive, bulky, and more complicated to use than some other non-invasive technologies. Additionally, the vast amounts of data acquired involve a significant amount of time and require technical skills to develop procedures for image analysis. Another potential issue concerns the health and safety concerns that may arise from equipment usage. However, these drawbacks create novel research opportunities in improving the time required for data acquisition whilst reducing data analysis to provide real-time characterization and defect detection. 

**Table 5:** Summary of the information concerning the application of X-ray micro-computed tomography in the evaluation of different processed products.

Products	Tube Voltage and Current	Spatial Resolution	Application	Reference
Banana slices	60 kV, 167 mA	15 µm	Effect of far-infrared radiation on the microstructure	[98]
Coffee beans	29 kV, 175 µm	2.8 µm	Microstructural changes induced by roasting	[100]
Coffee beans	19 and 20 keV	9 µm	Evaluation of microstructural properties	[99]
Minimally processed pomegranate arils	200 kV, 100 µA	71.4 µm	Characterization and estimation of pomegranate arils	[97]
Pomegranate juice	245 kV, 300 µA	71.4 µm	Characterization and estimation of pomegranate juice, aril, and peel	[96]
Pomegranate fruit parts	100 kV, 200 µA	71.4 µm	Estimation of pomegranate whole fruit and different parts	[94]

## 5. Raman Spectroscopy

### 5.1. Overview of Raman Spectroscopy

**Figure 5:**
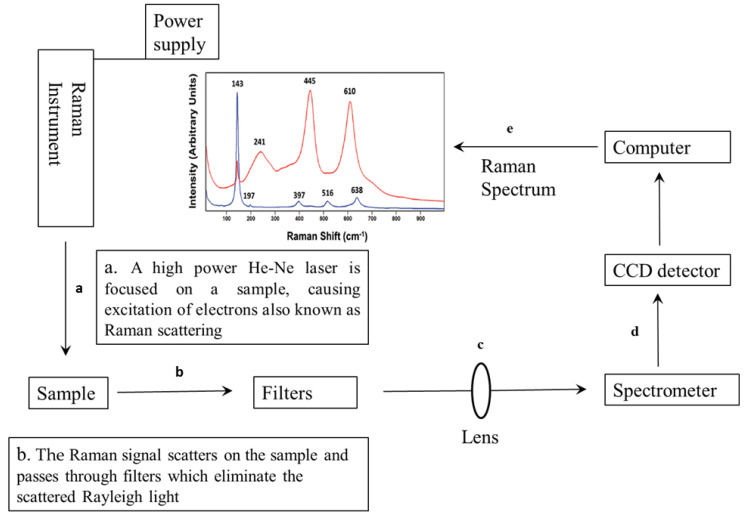
Block diagram illustrating basic steps using FT-Raman scattering. (**a**) high powered He-Ne laser provides an excitation signal to excite the sample of interest, (**b**) the Raman signal scatters on the sample and passed through filters which eliminate the scattered Rayleigh light and transmit the desired wavelength only, (**c**) the lens then focuses and transfers the scattering signals to the (**d**) Raman spectrometer which is detected by the CCD detector, (**e**) a computer analyses the signals to form a set of spatially offset Raman spectra.

Raman spectroscopy is another analytical vibrational spectroscopy that has been employed for food quality analysis and authenticity. Theoretically, Raman spectroscopy is based on the principle of interaction between light and the electron bonds within a molecule. Therefore, when light interacts with a molecule, it excites the electrons from a ground state into a virtual state before they relax into an excited vibrational state [101]. This scatter is referred to as Raman scattering. In this process, an elastic collision between the incident photon and the molecule of the sample occurs [102]. The scattered light is collected, dispersed in a monochromator, and then detected by a sensor [103]. There is a change in the rotational energy of the molecule, and the radiation causes a shift in the wavelength. The difference in frequency due to the incident radiation is called a Raman shift. When the molecule acquires energy, the photons can either shift towards longer wavelengths, also known as Stokes lines, or shorter wavelengths, known as anti-Stokes lines [14]. The spectra that are generated from this relaxation and excitation of molecules of samples by radiation of light impart a fingerprint region that can be used for structural and qualitative analysis. A block diagram illustrating the basic steps of Raman spectroscopy is presented in Figure 5.

Quantitative analysis can also be performed with Raman spectroscopy because there a linear relationship between the analyte band and the analyte concentration, and can be represented based on the following:

(1) Iv= IoKvC 

The term Iv reports the measured Raman intensity: Io is the excitation intensity, Kv is the constant, and C is the analyte concentration. Raman spectroscopy is another technique that uses vibrational spectroscopy and captures spectral information similar to the infrared spectrum, but they have some important distinctions. For instance, Raman spectroscopy provides a good signal-to-noise ratio compared to infrared spectroscopy, and it is highly specific and doesn’t overlap, making Raman spectroscopy ideal for fingerprinting samples.

Since water has a weak effect on the Raman spectra, Raman spectroscopy offers compatibility with the analysis of various aqueous samples, making it an ideal tool for analysing aqueous solutions. Advances in instrumentation, coupled with chemometric analysis, have led to Raman spectroscopy becoming a useful non-invasive technique in qualitative, quantitative, and structural analysis in the food and beverage industry. Raman spectroscopy has several advantages. It is rapid and requires little or no sample preparation, and is very compatible with aqueous solutions. These features make Raman spectroscopy an adaptable technique for in-line or online analysis in different fields of study, some of which include agricultural, pharmaceutical, biomedical, material science, geological, and environmental science [104].

### 5.2. Application of Raman Spectroscopy for Assessment of Processed Horticultural Products

#### 5.2.1. Dried Horticultural Products

Several studies on dried powder products have been performed on processed horticultural products (Table 6). Liang et al. [105] applied surface-enhanced Raman spectroscopy (SERS) to detect the adulteration of Rhodamine B in chilli powder. The authors were able to detect Rhodamine B in chilli powder and observed a limit of detection (0.08 mg/L), linearity (0.999), and precision (1.71–4.84%). Similarly, Pei et al. [106] discriminated between Sudan dye I and II in chilli powder and were able to detect to a limit of 0.6 mg/kg in the case of Sudan I and 0.4 mg/kg for Sudan II, respectively.

The presence of melanin yellow was successfully detected in turmeric powder within the frequency range of 1800–200 cm^−1^ [41]. The authors obtained a classification model with a prediction coefficient of determination of 0.916 and a limit of detection (LOD) of 1%. Li et al. [107] applied Raman spectroscopy to detect the presence of lead chrome green in tea powder at different concentrations. The models were developed using successive projections algorithm (SPA) and PLSR multivariate analysis. A successive projection algorithm was used for wavenumber selection and of the selected 8, the corresponding characteristic wavenumbers were 2775, 2176, 1666, 1541, 1297, 988, 547, and 262 cm^−1^. Based on these 8 characteristic wavenumbers, the detection model was built using PLSR. The authors reported that PLS models gave the best prediction statistics (*R*^2^ = 0.936, RMSEP = 0.803, LOD = 0.651 mg/g).

In another study, Ma et al. [108] applied SERS for authentication and detection of Carbendazim (CBZ) in tea. CBZ was detected with an accuracy of *R*^2^ = 0.954, residual standard deviation (RSD) = 12.4%, and an LOD limit of 0.1 mg kg^−1^.

#### 5.2.2. Juice Products

Several researchers have applied Raman spectroscopy for the evaluation of juice quality. A summary of Raman spectroscopy applied for the quality evaluation of horticultural juice products is presented in Table 7. For instance, Shende et al. [109] applied SERS with solid-phase extraction to orange juice to detect the presence of chlorpyrifos-methyl (CPM). The artificial addition of CPM to orange juice was detected in 12 min and at a concentration level of 50 ppb. For oranges, the observed concentration has been reported to be below the tolerance levels (0.1 ppm) for CPM. Malekfar et al. [110] reported the detection of carbohydrates and protein in tomato juice using SERS Raman spectroscopy. The authors applied a spectral range of 100 and 4000 cm^−1^, carbohydrates and protein were detected and assigned wavelengths of 738 cm^−1^, 1333 cm^−1^, and 2930 cm^−1^. The authors reported that Raman spectroscopy can be applied at control lines within the fruit processing industries. Nekvapil et al. [111] assessed the freshness of citrus juice using Raman spectroscopy within the spectral range (1800–100 cm^−1^) using two highly sensitive and portable systems. The authors reported that the coefficients of freshness (C_Fresh_) value for the three varieties of citrus juice (the values used to describe freshness) ranged between 2.8 to 3.5 for clementines (Day 1) and 1.5 for both mandarins and tangerines, respectively. 

#### 5.2.3. Oil Products

The application of Raman spectroscopy for the authentication, detection of adulteration, and evaluation of quality attributes of oil have been reported by various authors. For instance, El-Abassy et al. [115] applied Raman spectroscopy to develop calibration models using partial least squares regression. The study monitored the degradation of carotenoid content in extra virgin oil when heated by microwave and conventional processes in the spectral range of 1600–945 cm^−1^. Their developed model showed a high correlation coefficient *R*^2^ = 0.99 and low RMSE of 0.027 and 0.079 for calibration and prediction, respectively. Ahmad et al. [116] employed Raman spectroscopy to define the cooking range of extra virgin olive oil at 140–150 °C using a wavenumber range of 1800–540 cm^−1^. Sesame seed oil was assessed for authenticity and adulteration with other processed seed oils (e.g., vegetable oils from almond, castor, coconut, argan, avocado, macadamia, peanut, pumpkin, soybean, sunflower, olive carrot, jojoba, wheat seed, wild rose, marigold, and pomegranate). The authors reported that the developed models were able to discriminate to an accuracy of 99.83% for all the oil samples due to a specific spectral band at 1651 cm^−1^ associated with the C=C stretching mode [116]. Principal component linear discriminant analysis was applied, and sesame oil was successfully discriminated from other oil samples.

Raman spectroscopy is an emerging non-invasive technique for quality assessment and presents enormous potential for biochemical and chemical structural analysis. It is critically useful in situations where there is no need for sample preparation. One of the key advantages of Raman spectroscopy is its ability to provide information about the concentration, structure, and interaction of biochemical molecules within intact cells and tissues [117]. However, the applications of Raman spectroscopy in oil quality evaluation are mainly focused on fat content, lipid oxidation, and protein structures. As a single-point spectroscopic technique, it is more efficient for adulteration detection and quality assessment for homogeneous samples. In most heterogeneous food samples, such as in powdered products, a combination of Raman and HSI is preferably applied for analysis.

## 6. Nuclear Magnetic Resonance (NMR)

### 6.1. Overview of Nuclear Magnetic Resonance

**Figure 6:**
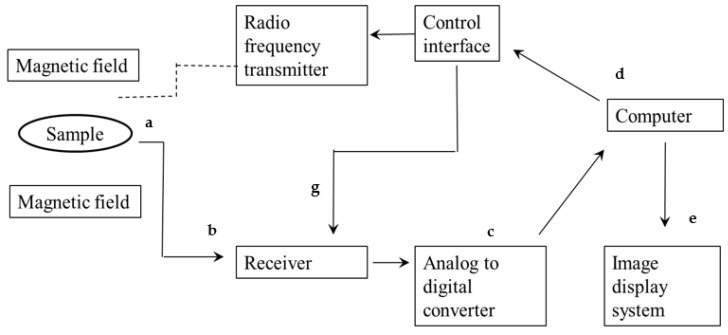
Block diagram of NMR imaging process. (**a**) very strong magnetic fields are generated using superconducting electromagnets, (**b**) the radio frequency input signal receiver collects input from the response of the sample (**c**–**g**); the signal produced by the interaction of the sample and magnetic field is not ready for interpretation and needs to be detected and processed to provide useful information, which is then transmitted to (**d**) a computer and (**e**) produces an NMR spectrum.

Nuclear magnetic resonance (NMR) was first discovered in the late 1930s by the scientist Isidor Rabi, when he successfully detected the magnetic spin of atomic nuclei in gases [118]. Today, NMR is widely used as a non-invasive technique for the structural analysis of organic materials [118]. A block diagram illustrating the basic steps of the NMR technology is presented in Figure 6.

Depending on the type of NMR application, NMR can be divided into three categories. These include magnetic resonance spectroscopy (NMR), magnetic resonance imaging (MRI), and low-field (LF) NMR [119]. NMR is based on the principle that nuclei have a spin and that all nuclei are electrically charged when exposed to a magnetic field and electromagnetic pulse. Therefore, when an external magnetic field is applied, a transfer of energy occurs at a specific wavelength range that corresponds to radio frequencies [76]. One of the most important applications of the NMR technique is to measure water content and water distribution due to certain elements, especially hydrogen nuclei, which show a high response to magnetic fields. NMR has unique advantages in detecting variations in the concentration or state of water and fats in fruits and vegetables. This derived information can be useful for assessing ripeness, defects, or decay in fruits and vegetables. NMR instruments require high magnetic fields and sophisticated electronics, so they are generally large and may be very expensive [120].

A summary of nuclear magnetic resonance applications in quality assessment for horticultural products is presented in Table 8. A review of the literature showed that NMR is mainly affected by the presence of water in food matrixes. A recent literature search indicates that much of the applications of NMR to processed products have been deployed in measuring the quality of whole fruits and liquid samples, such as fruit juices and oil samples.

### 6.2. Application of Nuclear Magnetic Resonance to Assessment of Processed Horticultural Products

#### 6.2.1. Juice Products

Several authors have reported applications of NMR with fruit juices. Flores et al. [122] applied time-domain nuclear magnetic resonance (TD-NMR), coupled with chemometrics, to predict different quality attributes in orange juice. The authors reported the standard error of prediction (SEP) for TSS (0.88) and pH (0.71). For sensitivity and selectivity, the author reported a prediction rate of 0.81 and 0.90, respectively, indicating that the method developed is highly suitable for the classification of oranges.

Caroline et al. [125] applied the ^1^H NMR and used a frequency of 400.13 MHz to detect adulteration in grape juice. Their model successfully detected the addition of other juices, achieving a classification accuracy of 0.93%. Vigneau et al. [123] applied the ^1^H NMR at 400 MHz for the qualitative evaluation of orange juice. The authors discriminated between authentic and adulterated (clementine juice) samples of orange juices. A total of 150 samples were taken, including both authentic and adulterated orange juices ranging in different concentrations (10–60%). The authors applied PLSR, principal components regression (PCR), and GA-PLS on the obtained NMR spectral data and reported that the GA-PLS algorithm provided the best performing model with an *R*^2^ of 0.79. In a study to discriminate between five mango cultivars, Koda et al. [121] applied ^1^H NMR on mango juice. The authors obtained spectra by band-selective excitation and successfully applied a PCA to the selective spectra. The authors identified several minor components in mango juice and assigned a signal to the minor components, including arginine, histidine, phenylalanine, glutamine, shikimic acid, and trigonelline. The results of their study showed that these minor components played a significant role in the classification and discrimination of the five different cultivars.

#### 6.2.2. Oil Products

NMR has been successfully applied for quality control of oil products derived from horticultural produce (Table 9). Skiera et al. [126] applied ^1^H-NMR to 120 samples of blends of different edible oils to determine the acid value and peroxide value. The authors used a frequency of 400.17 MHz and reported a relative sensitivity of 0.90%. Their model indicated that both methods (Classical and ^1^H-NMR) exhibited a similar analytical performance. Andrade et al. [127] assessed the presence of fatty acid methanol esters in different vegetable oil blends (e.g., soybean, corn, sunflower, canola, linseed, cottonseed, and jatropha) within a spectral wavelength of 200 MHz. The authors reported a chemical shift that is characteristic of the methoxyl groups in methyl esters. Their findings showed the limitation of ^1^H-NMR in the characterization of fatty acid methanol ester products and its success, as it obtained a good resolution for all the ^1^H-NMR spectra of the transesterification products.

Sega et al. [128] observed the ozonation of sesame oil using NMR. In their study, they established a relationship between the integral values of the signals corresponding to protons that resonate at either 5.29 and 1.97 ppm, or 5.11–5.08 ppm in the ^1^H-NMR for iodine and peroxide values, respectively. Table 9 provides a summary of NMR applied to the quality evaluation of processed horticultural oil products.

**Table 9:** Summary of nuclear magnetic resonance applied for quality evaluation of horticultural oil products.

Products	Parameters	Wavelength Range	Predictor’s Accuracy	References
EVOO	Stability of oil	300 MHz	Order of stability are MO > EVOO > AKO > SO.	[129]
Different blend of edible oil	Free fatty acid	400.17 MHz	Relative sensitivity = 0.90%	[126]
Different blends of vegetable oils	SFA, linoleic acid	200 MHz	methoxyl (δ = 3.70) and glyceryl methylene (δ = 4.10–4.40) protons, respectively.	[127]

EVOO, extra virgin olive oil; *R*^2^, coefficient of determination; SFA, saturated fatty acids; δ, chemical shift; MO, moringa oil; AKO, apricot kernel oil; SO, sunflower oil.

## 7. Other Spectroscopy Technologies

### 7.1. Dielectric Spectroscopy

#### 7.1.1. Overview of Dielectric Spectroscopy to Assessment of Processed Horticultural Products

Dielectric spectroscopy is another non-invasive technological tool useful for examining the interaction between the electric field and tested material. It derives from the effect of dielectric mechanisms and the polarization effect, which comprise the dielectric permittivity of the object. The dielectric properties of a binary mixture of solids and liquids have been researched extensively [130]. Dielectric spectroscopy uses electromagnetic fields and provides information on the dielectric response of materials. This technique is especially useful in evaluating the moisture content of different foods [131]. For most applications, the dielectric constant (ε′) and loss factor (ε″) are the studied properties of food materials. The dielectric constant indicates the material’s ability to store electric energy, whilst the loss factor is associated with energy dissipation or the conversion from electric energy to heat energy. Several variables may influence the dielectric properties of materials. These include the frequency of the applied alternating electric field, moisture content, bulk density, temperature, ionic nature, concentration (density), structure, and constituents of materials [132]. Dielectric properties usually correlate to temperature and frequencies, and these findings have been reported for different agricultural commodities.

#### 7.1.2. Application of Dielectric Spectroscopy to the Assessment of Processed Horticultural Products

The research literature suggests that the dielectric properties of agricultural products could be associated with their internal characteristics [133]. For instance, Lizhi et al. [130] researched the quantitative determination of adulterant levels in extra-virgin olive oil. Their results showed good prediction capability for the concentration of the vegetable oil adulterant on the olive oil. PCA was successfully used to classify the adulterant, while the PLS model showed good prediction statistics (*R*^2^ = 0.967, root mean square (RMS) = 0.053, LOD < 5%). For mangoes, Sosa-Morales et al. [134] evaluated the usage of an impedance analyser coupled with an open-ended coaxial-line probe to test the moisture content, soluble solids, pH, and electrical conductivity over 16 days. The authors observed that a frequency range of 10 MHz to 1.8 GHz at 25 °C resulted in a decrease dielectric constant (ε′) and loss factor (ε″) with time; this was caused by a reduction in moisture content and increase in pH during storage.

### 7.2. Fluorescence Spectroscopy

#### 7.2.1. Overview of Fluorescence Spectroscopy to Assessment of Processed Horticultural Products

Fluorescence spectroscopy is another emerging non-invasive technique that can be used to study the molecular structure and characteristics of foods. Currently, the development of commercial instrumentation, particularly front-face fluorescence spectroscopy (FFFS) and synchronous fluorescence spectroscopy (SFS), has gained traction in the quality analysis of food products. In fluorescence spectroscopy, the molecule exposed to fluorescence absorbs ultraviolet or visible light; after absorption, the fluorescent molecule is called a fluorophore [135]. Once the fluorophore absorbs energy from ultraviolet or visible light, the molecule releases energy in the form of the emission of light at a higher wavelength.

In theory, electrons of molecules may exist in several vibrational states. Electrons can be excited through ultraviolet or visible light, increasing their vibrational state, meaning that an electron goes from the ground singlet state, S_0_, to an excited singlet state, S_1_. When this occurs, the molecules absorb energy and emit it in the form of long wavelengths. Therefore, fluorescence spectroscopy can characterize molecules through excitation and emission spectra. The spectrofluorimeter, which is the instrument for measuring steady-state fluorescence, consists of the following components: A light source—this is usually a xenon or mercury lamp. Then two monochromator(s) and/or filter(s), one for selecting the excitation wavelengths and the other for selecting the emission wavelengths. Other components include a sample compartment, a detector that converts the emitted light to an electric signal, and a data acquisition and analysis unit.

#### 7.2.2. Application of Fluorescence Spectroscopy to Assessment of Processed Horticultural Products

Recently, fluorescence spectroscopy, in combination with multi-dimensional multivariate techniques, has been applied to evaluate food, dairy, and vegetable products. For instance, Boubellouta & Dufour [136] reported using synchronous fluorescence spectroscopy for determining the melting characteristics of two cheese varieties (Comté and Raclette). Saito [137] showed that laser-induced fluorescence spectroscopy could distinguish between normal and rotten Napa cabbage (*Brassica rapa* L.). The authors observed that the rotten core’s peak area (450 to 600 nm) was double that of the normal core.

Although this review focuses on applying fluorescence spectroscopy on processed horticultural products, the principles are broader, and fluorescence could be applied to fruits and vegetables and in other fields (e.g., pharmaceutical, biotechnology). Fluorescence spectroscopy is a very sensitive tool and is limited to samples or materials with a fluorescence effect [137]. Traditional right-angle fluorescence spectroscopic techniques cannot be applied to thick substances due to the large absorbance and effect of the scattering of light. The FFFS and SFS are two fluorescence spectroscopy methods that have been utilized to determine the quality characteristics of processed food substances with thick surfaces. However, with improved instrumentation, parts, and faster integration times, future analytical instrumentation that uses fluorescence spectroscopy could be a reliable non-invasive technique for studying molecular food structure and, consequently, their quality characteristics.

## 8. Future Prospects

The literature review suggests that infrared spectroscopy has been shown to be suitable for the quality assessment of oils, juices, and powders in the food and beverage industry. Infrared spectroscopy combined with advanced chemometric software packages has been applied successfully for on-line and in-line analysis, including the development of portable hand-held devices, making it the preferred method for non-invasive measurement. Compared to other non-invasive techniques, infrared spectroscopy has relatively inexpensive instrumentation costs, provides rapid and accurate assessment, and can allow for continuous on-line monitoring using fibre optics. HSI, on the other hand, is a visualization technique that offers high spatial and spectral resolution. Considering that limited information exists on the application of HSI and MSI to the quality evaluation of horticultural powders and liquids, future research with HSI and MSI should focus on developing models to predict various quality parameters, including detection adulteration. Furthermore, the development of future non-invasive systems for commercial implementation requires research to focus on reducing the total volume of the data, which is the key to building effective HSI and MSI systems. In practice, this means acquiring images with relatively low spatial resolution at a few important wavelengths; this would increase the speed of spectral data capturing and analysis. Currently, acquiring spatial and spectral data using HSI systems results in the accumulation of large multi-dimensional datasets, thus the application of HSI is mainly used for academic research.

On the other hand, NMR is best suited for the measurement and evaluation of food and processed agricultural products with high water content and water distribution, such as juices. This is due to certain elements being present, especially hydrogen nuclei from water molecules which show a high response to magnetic fields. The disadvantages of NMR systems, that prevent them from being used outside the research field, are that they require high magnetic fields and sophisticated electronics and, therefore, are generally quite large and expensive. Further research into the fabrication of small, easy-to-use, and cost-effective hand-held NMR devices is encouraged [138].

Compared to other non-invasive techniques, Raman spectroscopy can be applied to food substances in the form of solids or liquids and can be used to analyse gasses. Raman spectroscopy requires no prior sample preparation and can be applied regardless of sample thickness, shape, or size. It provides information about the concentration, structure, and interaction of biochemical molecules within intact cells. However, when comparing Raman spectroscopy to infrared spectroscopy, Raman spectroscopy has a low sensitivity for detecting concentrations for substances. In addition, Raman spectroscopy involves the heating of samples using a laser. This means that samples placed on metals and alloys cannot be analyzed. However, one of the disadvantages of both FT-IR spectroscopy and Raman spectroscopy is that they acquire spectral data from a single point of a sample, indicating that they may be better suited for inspection of authenticity and adulteration of processed food products. However, imaging techniques such as X-ray µCT are based on detection in variation in material density and, therefore, may be more suited to test for and detect defects within processed horticultural products [1]. Each non-invasive technique that has been discussed within this review has several advantages or limitations compared to the others. For example, infrared spectroscopy cannot detect compounds such as minerals, as these compounds are not active in the infrared region and do not produce any absorption bands [14]. However, Raman spectroscopy and NMR can measure the structural characteristics and quantify the mineral composition of foods. Therefore, a combination of different types of instrumentation may be applicable for quality assessment within the food and processing industry. Future research and design should focus on developing and manufacturing low-cost portable instrumentation with more robust algorithms and faster integration times. Through continuous improvement in measuring devices and chemometrics, these limitations are gradually being eliminated, thereby improving the commercial adoption of non-invasive technology.

## 9. Concluding Remarks

This review has highlighted the current potential of various non-invasive technologies for successfully assessing quality attributes related to processed horticultural products, such as juices oils and powders. Several of the non-invasive methods that have been reviewed within this paper are suitable for predicting the physical and chemical characteristics of processed products. The diverse chemometric analysis methods can be combined with various non-invasive techniques to extract relevant information from different processed food samples. However, the adoption of these non-invasive techniques for commercial grading and sorting is limited by several challenges associated with each technique. For instance, the high equipment cost required for setup, the significant time required for data acquisition and processing using HSI and X-ray CT systems, and the technical knowledge of these techniques, and skills required for data analysis and interpretation, limits their application [76]. Infrared technology, particularly near- and mid-infrared spectroscopy, has successfully measured the physical and chemical properties of oils, juices, and powders for both laboratory and industrial application. Further improvements to achieve more robust algorithms, cheaper, portable instrumentation, and faster integration times could improve its commercial adaptability. For imaging techniques such as HSI, MSI, and X-ray CT systems, future research needs to emphasise improving data acquisition, processing times, and making smaller, less expensive devices that can be adopted for real-time measurements at processing facilities. The adoption of these technologies by the industry will not only reduce the overall time and cost associated with conventional analysis but improve the quality control process of processed horticultural products.

## Figures and Tables

**Table 3:** Summary of infrared spectroscopy applied for quality evaluation of different horticultural oil products.

Products	Non-Invasive Method	Regression Analysis	Parameters	Wavelength Range	Predictors Accuracy	References
Extra virgin olive oil	NIRs	PLS	TSC SFA MUFA PUFA	9403–749 cm^−1^ 6800–6098, 5450–4597 cm^−1^ 5450–4597 cm^−1^ 9403–7498, 5025–4597 cm^−1^	*R*^2^ = 0.839, RPD = 2.64 *R*^2^ = 0.998, RPD = 21.8 *R*^2^ = 0.997, RPD = 18.7 *R*^2^ = 0.998, RPD = 25.1	[70]
Olive oil	ATR-FT-MIRs	PLS	MUFA PUFA SFA PV	3033–700 cm^−1^ 3033–700 cm^−1^ 3033–700 cm^−1^ 4000–700 cm^−1^	*R*^2^ = 0.89, REP = 1% *R*^2^ = 0.98, REP = 4% *R*^2^ = 0.71, REP = 6% *R*^2^ = 0.99, REP = 20%	[68]
Olive oil	NIRs Vis/NIRs	PLS	Squalene Squalene	350–2500 nm 1100–2300 nm	*R*^2^ = 0.83, RPD = 2.31 *R*^2^ = 0.74, RPD = 1.94	[69]
Virgin olive oil	NIRs	PLS	SFA PV TPC	12,500–4000 cm^−1^	*R*^2^ = 0.42, RPD = 1.13 *R*^2^ = 0.79, RPD = 1.64 *R*^2^ = 0.79, RPD = 1.71	[5]
Virgin coconut oil	ATR-FT-MIRs		PV	4000–650 cm^−1^	*R*^2^ = 0.982, RMSEP = 0.497	[64]
Virgin coconut oil	ATR-FT-MIRs		FFA	1730–1690 cm^−1^	*R*^2^ = 0.928, RMSEP = 0.126	[65]
Rapeseed and canola oil blend	NIRs	PLS	AV TPC	1800–2200 nm 1100–1800 nm	*R*^2^ = 0.99, RPD = 12.8 *R*^2^ = 0.98, RPD = 7.8	[67]
Palm and canola oil blend	NIRs	PLS	IV FFA PV	9404–7498 cm^−1^ 7502–6098 cm^−1^ 6102–5446 cm^−1^	*R*^2^ = 0.98, RPD = 6.11 *R*^2^ = 0.99, RPD = 11.60 *R*^2^ = 0.97, RPD = 6.40	[66]

PLS, partial least square; *R*^2^, coefficient of determination for validation; REP, relative error of prediction; FFA, free fatty acid; IV, iodine value; AV, acid value; PV, peroxide value; TSC, total sterol content; SFA, saturated fatty acid; MUFA, monounsaturated fatty acid; PUFA, polyunsaturated fatty acid; TPC, total phenolic content; NIRs, near-infrared spectroscopy; Vis/NIRs, visible to near-infrared spectroscopy; ATR-FT-MIRs, attenuated total reflectance Fourier transform mid-infrared spectroscopy.

**Table 4:** Application of hyperspectral imaging in the evaluation of different processed horticultural products.

Product	Non-Invasive Method	Regression Analysis	Parameters	Wavelength Range	Predictors Accuracy	Reference
Coffee beans	HSI	PLS	Aroma compounds	1000–2500 nm	*R*^2^ = 0.21–0.71, RPD = 0.84–1.87	[84]
Tea	HSI	PLS	Polyphenols	405–970 nm	*R*^2^ = 0.915	[89]
Tomato seed	MSI	PLS-DA	Variety discrimination	375–970 nm	Classification = 94–100%	[81]
Tomato seed	HSI	PLS-DA	Variety discrimination	375–970 nm	≥82%	[80]
Spinach seed	HSI	PLS-DA	Germination ability	395–970 nm	68%	[82]
Nutmeg powder	HSI	PCA, ANN and PLS-DA	Spent powder	400–1000 nm	*R*^2^ = 0.98, LOD = 5%	[90]
Virgin olive oil	HSI	PLS GA-PLS	Acidity, Peroxide value, Humidity content Acidity Peroxide value humidity content	900–1700 nm 900–1700 nm	*R*^2^ = 0.95 *R*^2^ = 0.98 *R*^2^ = 0.91 *R*^2^ = 0.93 *R*^2^ = 0.92 *R*^2^ = 0.92	[88]
Watermelon seeds	HSI	PLA-DA	Virus infection	1411–1867 nm	83.3%	[83]
Cooking oil blend	HSI	PLS	Classification	350–2500 nm	100%	[87]

PLS, partial least square; PLS-DA, partial least square discriminant analysis; ANN, artificial neural networks; GA-PLS, genetic algorithm-partial least square; *R*^2^, coefficient of determination for validation; HSI, hyperspectral imaging; LOD, limit of detection.

**Table 6:** Summary of Raman spectroscopy applied for quality evaluation of powdered products.

Products	Parameters	Wavelength Range	Multivariate Analysis	Predictors Accuracy	References
Chilli powder	Sudan I dye adulterant	2000–200 cm^−1^	SG, SNV, PCA, PCR, PLS-DA	*R*^2^ = 0.891–0.994	[46]
Chilli powder	Sudan I, Sudan II adulterants	1700–400 cm^−1^	PCA	Detection of 0.6 mg/kg and 0.4 mg/kg for Sudan I and II, respectively	[106]
Turmeric powder	Melanil yellow	3700–100 cm^−1^	SG, MSC, BR	LOD = 1%	[41]
Tea powder	Lead chrome green	2804–230 cm^−1^	PLSR, SPA	*R*^2^ = 0.858	[107]
Chilli powder	Rhodamine B	1800–200 cm^−1^	–	Linearity = 0.999, LOD = 0.08%	[105]
Paprika powder	Sudan I adulterant	2200–200 cm^−1^	PCA, PLSR	*R*^2^ = 0.788–0.983	[36]

PLS, partial least square; PLS-DA, partial least square discriminant analysis; PLSR, partial least square regression; LOD, limit of detection; PCA, principal component analysis; PCR, principal components regression; SPA, successive projections algorithm; SNV, standard normal variate; SG, Savitzky–Golay; MSC, multiplicative scatter correction; BR, band ratio.

**Table 7:** Summary of Raman spectroscopy applied for quality evaluation of horticultural juice products.

Products	Parameters	Wavelength Range	Predictors Accuracy	References
Apple juice	Detection of phosmet concentration in standard apply	2000–200 cm^−1^	*R*^2^ = 0.905–0.984	[112]
Citrus juice	Degree of freshness	1800–100 cm^−1^	C_fresh_ range from 2.8 to 3.5	[111]
Pear juice	Detection of *A. alternate*	1800–400 cm^−1^	LOD = 1.0 × 10^3^ cfu/mL	[113]
Tomato juice	Carbohydrates, protein	3900–400 cm^−1^	738 cm^−1^, 1333 cm^−1^ and 2930 cm^−1^ assigned to carbohydrates	[110]
Orange juice	Chlorpyrifos-methyl (CPM)	1800–400 cm^−1^	LOD = 50 ppb	[109]
Carrot juice	Polyacetylenes, carotenoids	2300–200 cm^−1^	LOD = 1400 μg/g	[114]

Cfu, colony-forming unit; C_fresh;_, coefficient of freshness; LOD, limit of detection; PLSR, partial least square regression; PPB, parts per billion; SGS, Savitzky–Golay smoothing.

**Table 8:** Summary of nuclear magnetic resonance spectroscopy applied for quality evaluation of horticultural juice products.

Products	Parameters	Frequency Range	Multivariate Analysis	Predictor’s Accuracy	References
Mango juice	Discrimination of different cultivars	0.8 MHz	PCA	LOD = 3.0–5.5 ppm	[121]
Orange juice	TSS pH	8.5 MHz	PLSR, S-GA	SEP = 0.88 SEP = 0.17	[122]
Orange juice	Discrimination of pure and adulterated orange juice	400 MHz	PLSR, PCR, GA-PLS	*R*^2^ = 0.79	[123]
Pomegranate juice	TA TSS pH	1.7 MHz	PLS	*R*^2^ = 0.54 *R*^2^ = 0.60 *R*^2^ = 0.63	[124]

PCA, principal component analysis; PCR, principal component regression; PLSR, partial least square regression; GA-PLS, genetic algorithm-partial least square; PPM, parts per million; LOD, limit of detection; SEP, standard error of prediction; *R*^2^, coefficient of determination; TSS, total soluble solids; TA, titratable acidity.

## Data Availability

Not applicable.
